# An Investigation to Study the Effect of Process Parameters on the Strength and Fatigue Behavior of 3D-Printed PLA-Graphene

**DOI:** 10.3390/polym13193218

**Published:** 2021-09-23

**Authors:** Anouar EL MAGRI, Saeedeh VANAEI, Mohammadali SHIRINBAYAN, Sébastien Vaudreuil, Abbas TCHARKHTCHI

**Affiliations:** 1 Euromed Polytechnic School, Euromed Research Center, Euromed University of Fes, Route de Meknès (Rond point Bensouda), Fès 30 000, Morocco; s.vaudreuil@ueuromed.org; 2 GREMAN, INSA Centre Val de Loire, 41000 Blois, France; saeedeh.vanaei@ensam.eu; 3 Arts et Metiers Institute of Technology, CNRS, CNAM, PIMM, HESAM University, 75013 Paris, France; mohammadali.shirinbayan@ensam.eu (M.S.); abbas.tcharkhtchi@ensam.eu (A.T.)

**Keywords:** 3D printing, fatigue, damage, PLA-Graphene, inter-layer bonding

## Abstract

3D printing, an additive manufacturing process, draws particular attention due to its ability to produce components directly from a 3D model; however, the mechanical properties of the produced pieces are limited. In this paper, we present, from the experimental aspect, the fatigue behavior and damage analysis of polylactic acid (PLA)-Graphene manufactured using 3D printing. The main purpose of this paper is to analyze the combined effect of process parameters, loading amplitude, and frequency on fatigue behavior of the 3D-printed PLA-Graphene specimens. Firstly, a specific case study (single printed filament) was analyzed and compared with spool material for understanding the nature of 3D printing of the material. Specific experiments of quasi-static tensile tests are performed. A strong variation of fatigue strength as a function of the loading amplitude, frequency, and process parameters is also presented. The obtained experimental results highlight that fatigue lifetime clearly depends on the process parameters as well as the loading amplitude and frequency. Moreover, when the frequency is 80 Hz, the coupling effect of thermal and mechanical fatigue causes self-heating, which decreases the fatigue lifetime. This paper comprises useful data regarding the mechanical behavior and fatigue lifetime of 3D-printed PLA-Graphene specimens. In fact, it evaluates the effect of process parameters based on the nature of this process, which is classified as a thermally-driven process.

## 1. Introduction

Additive manufacturing (AM) is a family of processes enabling the layer-upon-layer production of an object from three-dimensional (3D) model data [1,2]. This process has shown significant potential and alternative methods to process materials for use in different industries [3,4,5]. One of the many benefits of AM is the production of functional parts with complex geometries that are difficult to manufacture by subtractive manufacturing methodologies [6,7].

Thanks to its ease of use and lower investment and operating costs, fused filament fabrication (FFF) is an extensively used AM technology [8]. In this method, the thermoplastic filament is fed into a heated nozzle, melted, and subsequently extruded and deposited layer by layer onto a build plate forming the desired 3D part [9,10]. Various thermoplastics and composites are currently used to produce 3D parts satisfactorily by the FFF process [11,12,13]. In the latter, multiple controllable printing parameters, such as liquefier and platform temperature, print speed, raster angle, and layer thickness, are accounted to produce highly qualified 3D-printed parts. Most studies reported the dependence between the mechanical properties and the selected printing parameters [14,15,16,17,18,19,20]. Besides, there are numerous research projects on thermoplastics such as polycarbonate (PC), acrylonitrile butadiene styrene (ABS), and polylactic acid (PLA) to estimate and analyze their mechanical properties and especially fatigue analysis.

Accordingly, several studies were performed in consideration of the effect of process parameters on the 3D-printed structures [21,22,23]. El Magri et al. investigated the effect of liquefier temperature and infill orientation on tensile properties and crystallinity of 3D-printed virgin polylactic acid (PLA) and short carbon fiber (CF)-reinforced PLA. For both materials, the maximum tensile properties have been attained for T_Liq_ = 230 °C and (0/15/−15°) infill orientations. In another study, researchers found that the liquefier temperature, print speed, raster angle, and layer thickness parameters need to be taken into serious consideration. They found that these printing parameters impact directly the tensile properties and crystallinity of FFF printed materials [2,8,11]. Another study performed to determine the tensile properties and surface roughness of ABS to optimize the process parameters of FFF [24]. Similarly, a work on PC specimens was implemented to analyze the tensile strength of the 3D-printed parts. Compared with the extruded PC, they observed approximately 75% enhancement in tensile properties [25].

With reference to the mentioned works, FFF could offer enhanced fatigue lifetime of the final part in comparison with the traditional manufacturing processes [26,27,28]. The effect of print orientation on fatigue lifetime of 3D-printed ABS samples has been considered by Lee and Huang [29]. In addition, PLA, as a known material in the 3D printing process with a high mechanical strength, is stronger than ABS but with a lower ductility [29]. Its elevated mechanical properties make this material to be applicable in the 3D printing process [30]. In addition, besides the work performed on the mechanical properties of PLA as a composite [31,32], several studies investigated the mechanical performance of PLA considering FFF parameters [33,34,35,36,37].

Graphene’s addition to polymer matrices is currently under investigation as a promising method to improve their service conditions. Its excellent mechanical, electrical, and thermal properties make it an appropriate candidate for the reinforcement of several polymers [38,39]. Recently, PLA-Graphene nanocomposites have been fabricated using 3D printing process due to attractive features offered by both materials. However, in composites, the main challenge is to understand the graphene properties’ transfer from the nanoscale to the macroscale. Caminero et al. [38] reported that the printed PLA-Graphene composite samples showed the best performance in terms of surface texture, tensile, and flexural stress compared with un-reinforced PLA. However, the impact strength of the PLA-Graphene composite samples has been reduced by 1.2–1.3 times compared with that of un-reinforced PLA. By using central composite design, Camargo et al. [40] studied the effect of the variation of the infill and layer thickness parameters on the mechanical properties of 3D-printed PLA-Graphene. The results showed that the mechanical properties increase by the enhancement of the layer thickness and infill density parameters, while impact energy decreases as infill increases. Moreover, the coupled effect of the cyclic loading amplitude and frequency on the fatigue behavior of materials (especially composites) and the induced self-heating phenomenon have been studied [41,42]. It has been shown that, at high values of frequency and applied stress during the first stage of low cycles fatigue, composites exhibit an overall fatigue response mainly governed by the induced thermal fatigue (ITF). Accordingly, the mechanical fatigue (MF) nature becomes predominant during the second stage before the failure. For low frequency and applied amplitude, no significant self-heating phenomenon has been observed.

To conclude, there is still a lack of research on the development of this material. In this work, at the outset, the nature of the mechanical strength of 3D-printed specimens is discussed. Then, their fatigue behavior is evaluated by considering the condition of printing during the process. Finally, the relative Young’s modulus evolution and self-heating phenomenon are presented in order to have a better point of view through the fatigue lifetime of the 3D-printed specimens.

## 2. Material Description and Experimental Methods

### 2.1. PLA-Graphene Filament

In our study, we used a commercial PLA-Graphene filament (d = 1.75 mm). Some physico-chemical properties are presented in Table 1.

### 2.2. 3D Printer Device

An INTAMSYS (intelligent additive manufacturing systems), FunMAT HT (Shanghai, China) was used for processing PLA-Graphene. This FFF system, equipped with both a heated bed and build chamber, has a build volume of 260 × 260 × 260 mm^3^. Tensile test specimens were printed directly on the heated glass bed. The infill parameter was set to 100% to obtain solid-like samples. The major printing parameters evaluated are summarized in Table 2. All specimens were printed flat on the build platform (XY surface). Slicing of the 3D-model into individual layers was performed using the INTAM-suite software v3.5.2 (Shanghai, China).

### 2.3. Characterization Methods and Experimental Procedure

#### 2.3.1. Microscopic Observation

To investigate the material microstructure in 3D-printed samples, we implemented microscopic observations using a scanning electronic microscope (HITACHI 4800 SEM, Tokyo, Japan).

#### 2.3.2. Quasi-Static Tensile Test

Tensile tests until failure were carried out following ASTM D638–14 “Standard Test Method for tensile Properties of Plastics” (American technical standard) at room temperature on MTS 830 hydraulic machine (Eden Prairie, MN, USA) equipped with a 10 kN load cell and self-tightening jaws, which was used for all series of specimens.

**Figure 1:**
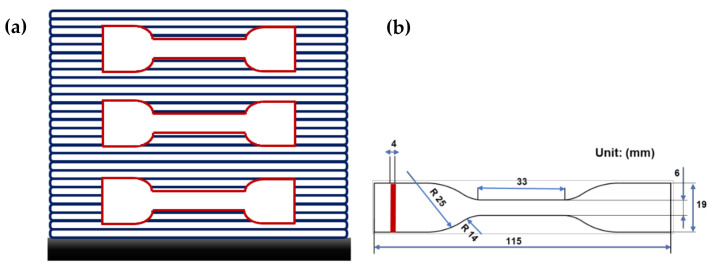

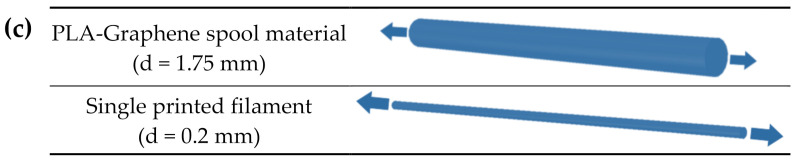
Representation of (**a**) location of samples on the printed solid blocks, (**b**) printed samples based on ASTM D638 type IV, and (**c**) proposed printed filaments to perform tensile test.

Firstly, the quasi-static tensile specimens were cut from the printed solid blocks (Figure 1a) using a proper standard mold and a press machine. Then, tensile test with a velocity of 5 mm/min was applied on the printed sample (based on ASTM D638 type IV in Figure 1b). Notably, a contactless technique (using a camera) is used to measure the local deformation parallel to the tensile test. Displacement (mm) and force (N) were collected and processed by the MTS Test Suite software (Eden Prairie, MN, USA) to establish tensile strain–stress curves and calculate the tensile properties (Young’s modulus; tensile strength and strain at break). Young’s modulus was determined from the slope of the obtained stress–strain curves. The tensile strength was calculated by dividing the maximum applied load by the cross-sectional area of the specimen. The strain at break was measured using non-contact extensometer. All the reported values were calculated as averages over five specimens. Additionally, tensile tests on spool material and single printed filament were proposed (Figure 1c). The objective of this step is to understand the effect of bonding and filament deposition on tensile behavior of the printed samples.

#### 2.3.3. Fatigue Test

Fatigue test (tension–tension mode) was carried out at different applied maximum stresses on MTS 830 hydraulic fatigue machine using the same standard as indicated in Figure 1 (ASTM D638 type IV). We chose the minimum applied stress to be equal to 10% of the maximum applied stress (R_σ_ = 0.1). In this paper, the results of the experimental tests at different frequencies of 10, 40, and 80 Hz are presented. During cyclic loading, as the temperature rises (due to the composite self-heating), the temperature variation was measured using an infrared camera (Optris PI450) in a specific area (maximum temperature). Material emissivity (ε) was obtained by calibrating the absolute difference of the tracks obtained by IR-camera and a thermocouple.

### 2.4. Condition of Printing

In this paper, we defined three conditions by considering platform temperature and print speed, as shown in Table 3. Five samples were tested for each condition of tensile tests.

## 3. Results and Discussion

In this section, the results of tensile test and tension–tension fatigue test were presented. During the sample preparation, the liquefier temperature was fixed at 200 °C for all conditions, and the samples were printed following the mentioned conditions. Furthermore, during the fatigue test, efforts were made to measure the evolution of Young’s modulus and also the self-heating caused during the fatigue test.

### 3.1. Quasi-Static Tensile Test

#### 3.1.1. PLA-Graphene Filament

**Figure 2:**
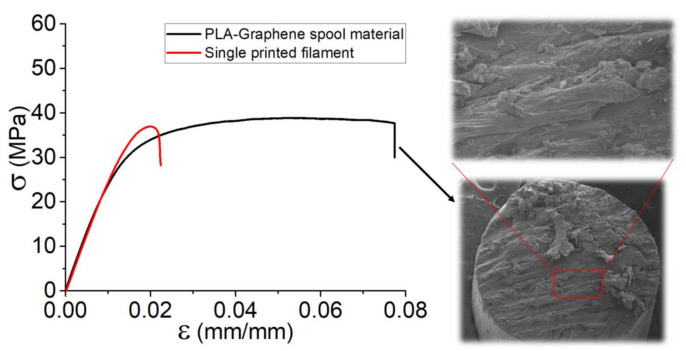
Tensile curves of spool material and single printed filament.

Based on Figure 2, to show the effect of layer-by-layer deposition, the results of tensile tests for the set of specimens were presented. Regardless of the variation in failure strain for the two specimens (PLA-Graphene spool material and one printed filament), there were no differences in Young’s modulus. However, the remarkable failure strain in the case of spool material is a notable issue in determination of the mechanical behavior of 3D-printed parts. It appears that, by extruding a filament through the extruder and consequently printing a single filament, the failure strain was decreased about 75%. In addition, regarding the brittle behavior of the material, the failure strain of PLA-Graphene spool filament (~7.5%) is almost 4 times greater than that of single printed filaments (~2%). This fact could be considered as a confirmation of the underlying assumption that in 3D printing and by extrusion of the filament through a liquefier, the mechanical behavior and particularly the elongation were markedly affected after deposition and printing. Moreover, in order to show the ductility of the PLA-Graphene spool material, cross-sectional morphology of the filament was observed right after the failure of the material, as included in Figure 2.

#### 3.1.2. Influence of Process Parameters on the Tensile Behavior of Printed Vertical Walls

**Figure 3:**
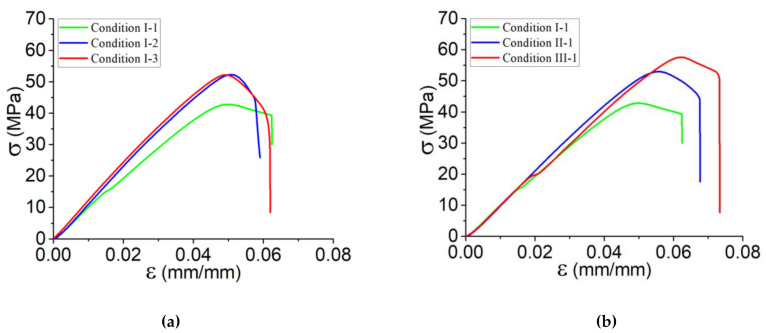
Tensile behavior for the set of samples cut from the printed solid blocks according to (**a**) conditions I-1, I-2, and I-3, representing the variation of platform temperature and (**b**) conditions I-1, II-1, and III-1, representing the variation of print speed.

Tensile behaviors of the set of samples cut from the vertical wall are illustrated in Figure 3, including the tensile behavior at different values of platform temperature and print speed. The results indicate that the ultimate strength increases when both platform temperature and print speed increase, in which case the influence of print speed is more significance. The highest ultimate strength refers to the samples of the printed solid blocks at V = 40 mm s^−1^, whereas the highest Young’s modulus belongs to those printed at T_platfrom_ = 60 °C. These observations refer to the fact that the decrease in cooling rate of PLA-Graphene by the increase in the platform temperature allows the material to have sufficient time for crystallization (above crystallization temperature) and re-arrangement of the polymer chains [36]. The same point exists for the variation of the print speed by which it helps again the deposited filaments not to be cooled down rapidly. Given the above-mentioned results and also the curves presented in Figure 3, the following statements are summarized:

- The influence of T_platform_ on ultimate strength is limited. When platform temperature is increased, the ultimate strength varies around 24%, whereas the increase in print speed causes approximately a 38% variation.
- Average failure strain occurs around 6% by variation of platform temperature, which is around 7% in the case of changing of print speed.
- In general, the variation of Young’s modulus in the case of the samples printed at different platform temperature is higher than that of a different print speed.
- Overall, print speed has more influence on the enhancement of the bonding and strength of the deposited filaments.

#### 3.1.3. Influence of Process Parameters on the Tensile Behavior of Printed Tensile Samples

**Figure 4:**
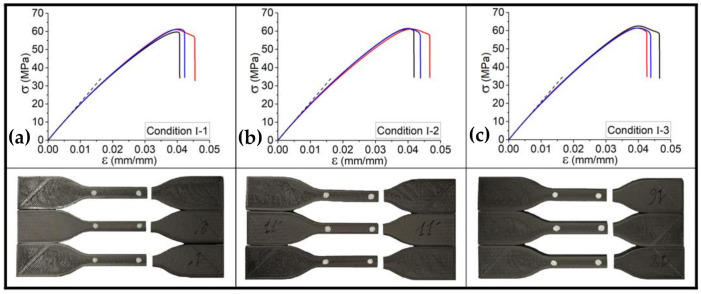
Tensile behavior for the set of printed samples according to conditions (**a**) I-1, (**b**) I-2, and (**c**) I-3, representing the variation of platform temperature. (The dash-lines show how the slope of the tensile curve varies).

Figure 4 illustrates the results of quasi-static tensile test on printed samples according to Condition I including the samples printed at various platform temperature as T_platform_ = 40, 50, and 60 °C. The value of Young’s modulus of the sample printed at T_platform_ = 40 °C is about 1.8 GPa, which is lower than those samples printed at T_platform_ = 50 °C and T_platform_ = 60 °C; however, the effect platform temperature variation is limited. On the other hand, the ultimate stress and consequently its strain gradually increase, and by increasing the platform temperature from 40 to 60 °C, a ~5% increase in ultimate stress was observed. Seemingly, these variations could be negligible; however, platform temperature has its own effect and causes a heat transfer through the deposited filaments. Another convincing point is the repeatability of the set of specimens by the occurrence of rupture at one edge of the samples as well as the fact that the failure mode was due to the material departure in a plane almost normal to the tensile stress. As expressed in Section 3.1.1, the failure strain of the material and brittle failure could be accordingly accomplished by the printed samples, which consist of deposition sequences of filaments (layer-by-layer deposition of filament). Notably, the mentioned deposition sequences contributed to a failure strain ~2.5 times higher than that of the printed single filament, which confirms the dependency of strength of the printed structures on the deposited layers.

**Figure 5:**
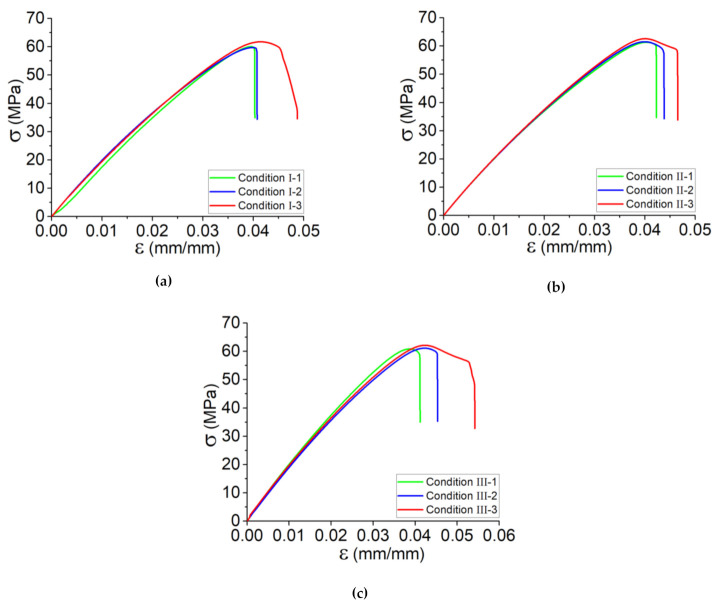
Tensile behavior for the set of samples according to the conditions (**a**) I, (**b**) II, and (**c**) III representing the variation platform temperature and print speed.

In order to have a better presentation on the characterization of the specimens, tensile tests were applied at least five times for each condition. Following the presented results in Figure 4, the accompanying graphs in Figure 5 and data collected in Table 4 indicate the tensile behavior for the set of five specimens assessed according to the defined conditions by investigating the influence of platform temperature (T_Platform_ = 40, 50, 60 °C) and print speed (V_liq_ = 20, 30, 40 mm/s). In these curves, the strong effect of the process parameters, samples cut from a vertical wall including single layers deposited on top of each other, as discussed in Section 3.1.2, is clearly emphasized in terms of Young’s modulus and inelastic behavior until failure. One can note that the tensile test, for each kind of specimen, has similar Young’s modulus in the first linear period. Moreover, except for the samples printed in the conditions with the highest platform temperature (conditions I-3, II-3, and III-3), tensile behavior appears to be similar until failure. As mentioned, the higher the platform temperature is, the better the crystallization occurs, resulting in a better bonding between deposited layers. According to the presented results in Figure 5, these overall results could be taken into account thus far:

- By increasing the print speed in the range of 20 mm/s–40 mm/s (e.g., conditions I-1 and III-1), the tensile strength of the PLA-Graphene increased by 5%.
- By increasing the print speed in the range of 20 mm/s–40 mm/s (e.g., conditions I-3 and III-3), the failure strain increased by 20%.
- By increasing the platform temperature from 40 °C to 60 °C in each condition group (e.g., condition I), Young’s modulus and tensile strength increased by 17% and 5%, respectively.
- By increasing the platform temperature from 40 °C to 60 °C in each condition group (e.g., condition II), the failure strain increased by 12%.

**Figure 6:**
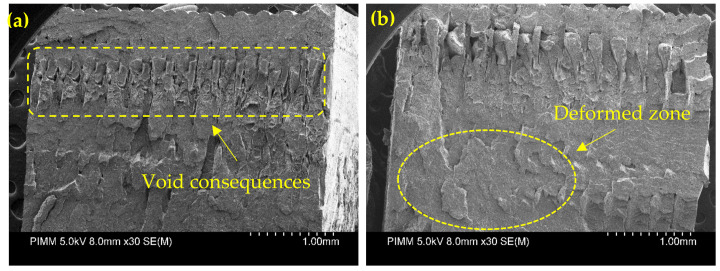
SEM micrograph for tensile fracture surface of the specimen in (**a**) condition III-1 and (**b**) condition III-3.

Figure 6 shows the SEM micrographs of a fractured sample of condition III-1 and condition III-3. According to the observations from tensile results and by considering the fact that there was a variation in the ductility of the material, it seems that the ductility is observable based on Figure 6b. As high platform temperature produces more energy and makes the deposited material difficult to solidify, by raising the temperature profile of the filaments during deposition, the adequate infiltration and diffusion among filaments could improve the inter-layer bonding through the deposited filaments [16].

### 3.2. Fatigue Behavior Analysis

**Figure 7:**
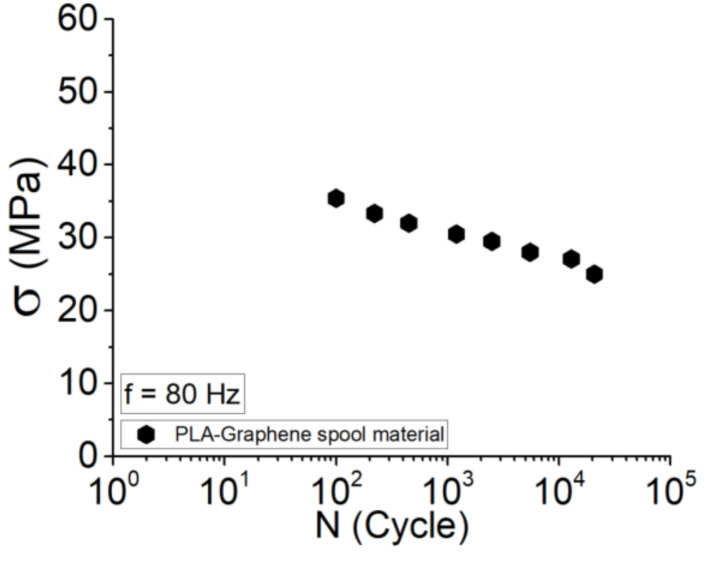
Wöhler curve for PLA-Graphene spool material (as-received filament).

Following the tensile behavior of the spool material (as-received filament), with reference to Figure 2, efforts were made to perform a tension-tension stress-controlled fatigue test on the as-received filament by defining a length equal to the printed dog-bone sample tests at the frequency of 1 Hz (see Figure 7). The Wöhler curve shows that, at the high amplitudes (~35 MPa), the fatigue life time is about 100 cycles, whereas a variation of 15% and then 40% in applied stress leads to fatigue life of 5000 and 2 × 10^4^ cycles, respectively. It could be established that the ductility observed for the spool material could lead to high life cycle, although at low strength.

In what follows, the life cycle, fatigue behavior, and the evolution of relative Young’s modulus as well as the self-heating of the printed dog-bone samples are discussed. It is worth mentioning to say that these samples were printed according to the conditions presented in Table 3.

#### 3.2.1. Influence of Process Parameters

**Figure 8:**
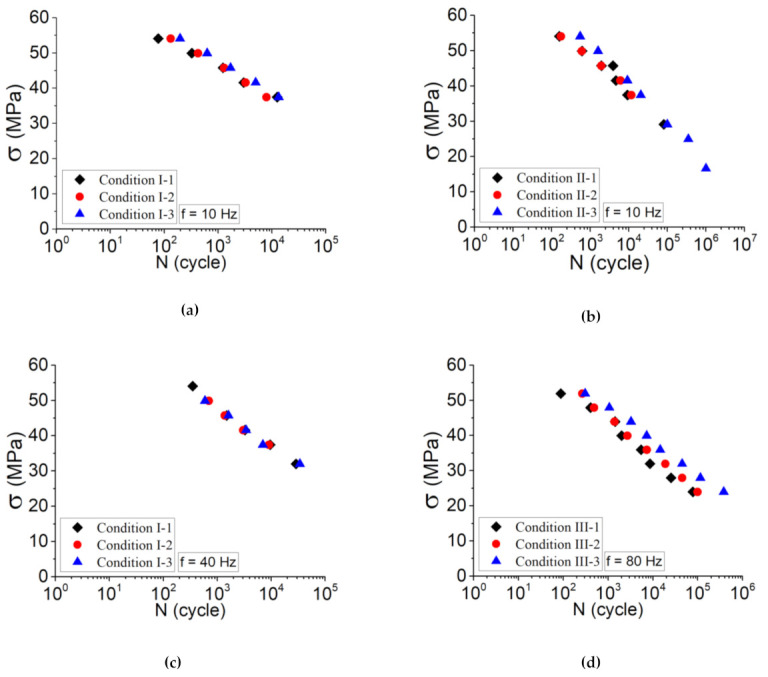
Wöhler curves for printed samples in tension–tension fatigue tests according to (**a**) condition I at f = 10 Hz, (**b**) condition II at f = 10 Hz, (**c**) condition I at f = 40 Hz, and (**d**) condition III at f = 80 Hz.

Figure 8 shows the Wöhler curves obtained in tension–tension stress-controlled fatigue tests, including the results of: (a) conditions I at f = 10 Hz; (b) conditions II at f = 10 Hz; (c) conditions I at f = 40 Hz; (d) conditions III at f = 80 Hz. The obtained results show that by variation of platform temperature, at both high and low amplitudes, there is a variation in the life cycle of the material. Based on the samples printed according to condition I (Figure 8a), a variation of 20 °C in platform temperature causes a fatigue lifetime of two times greater. In addition, the same observation was conducted in the case of samples printed according to condition II; however, the overall fatigue lifetime for these samples was raised by 10 times. It is worth mentioning to say that the effect of print speed on these comparisons is inevitable.

Comparing the samples printed as shown in Figure 8a,b, one can note that a variation of 10 mm s^−1^ of print speed (e.g., condition I-3 and II-3) leads to a fatigue lifetime four times greater. These observations affirm the influence of process parameters on the strength of the 3D-printed parts and thus their fatigue lifetime.

Following Figure 8c,d, the influence of frequency was taken into account by performing the tension–tension fatigue test at elevated frequencies. It has a determinant influence, independently of the loading amplitudes, on the fatigue lifetime of the material and particularly at high loading amplitudes. The main reason for this issue is the self-heating occurred during fatigue test. For the samples of condition I at the same amplitudes, tension–tension fatigue test at f = 40 Hz was carried out, and the corresponding Wöhler curves are shown in Figure 8c.

#### 3.2.2. Relative Young’s Modulus Evolution

**Figure 9:**
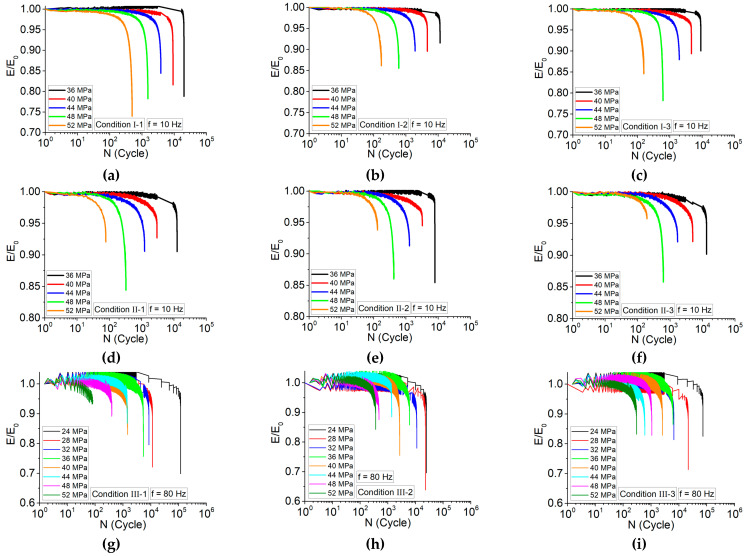
Evolution of the relative Young’s modulus (E/E_0_) during tension–tension fatigue tests: (**a**) condition I-1; (**b**) condition I-2, (**c**) condition I-3 at frequency of 10 Hz, (**d**) condition II-1, (**e**) condition II-2, (**f**) condition II-3 at frequency of 10 Hz, (**g**) condition III-1, (**h**) condition III-2, and (**i**) condition III-3 at frequency of 80 Hz.

Following the results of the fatigue test, the evolution of the relative Young’s modulus for three different conditions (in each amplitude) was illustrated in Figure 9 for the frequencies of 10, 10, and 80 Hz, respectively. Almost all samples exhibit a fatigue behavior mostly governed by the MF nature due to the damage phenomenon. However, the ITF is a predominant nature of the PLA-Graphene fatigue behavior at high amplitude. From these curves, at high loading amplitudes, the dynamic modulus decreases rapidly in a non-linear logarithmic regime until fracture of the sample. For low applied amplitudes, the dynamic modulus exhibits two decreasing regimes: a linear one during the initial cycles, and a drastic decrease just before the fracture. The following observations could be extracted:

- The higher the platform temperature, the lower the damage caused during fatigue test.
- The significance of kinetic damage is observed for the samples printed with the lowest platform temperature and print speed (in a same frequency).
- The higher the applied frequency, the higher the damage appeared during the fatigue test.

Given the above statements, one can mention that the mechanism of inter-layer bonding through the deposited layers improves with variation of parameters. In this case, it was mentioned that both high platform temperature and print speed decrease the cooling rate of the deposited filaments and allow the material to form a better bonding at the interface of the deposited layers.

#### 3.2.3. Self-Heating Phenomenon

The fatigue behavior of PLA-Graphene induces self-heating as a result of variation in loading conditions caused by implementation of different amplitude and frequency. The viscous behavior of the polymer as a function of the temperature enhancement depends on the material transition temperatures. Accordingly, the coupled effect of high loading amplitude and high frequencies generates more intensive self-healing and damage phenomena.

**Figure 10:**
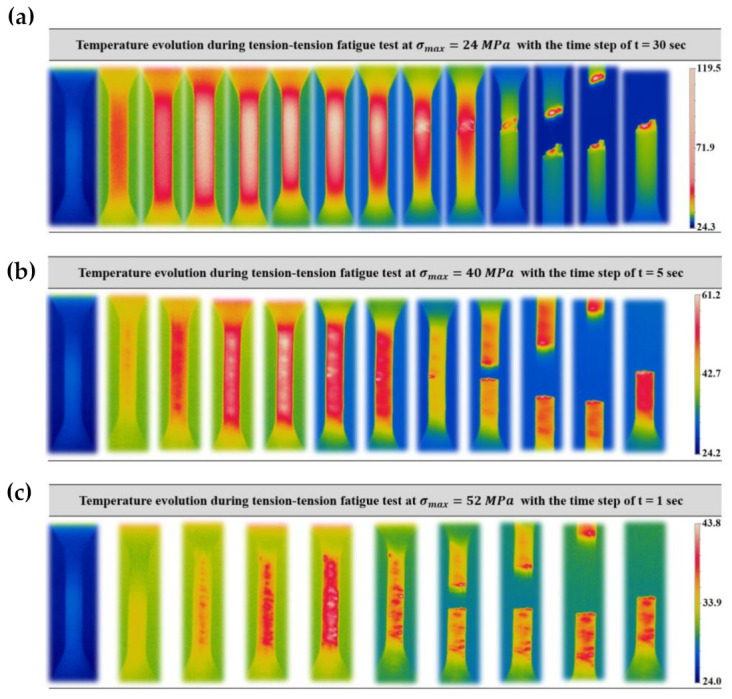
Induced temperature under tension–tension fatigue test for condition III-3 at frequency of 80 Hz: (**a**) low amplitude, (**b**) medium amplitude, and (**c**) high amplitude.

**Figure 11:**
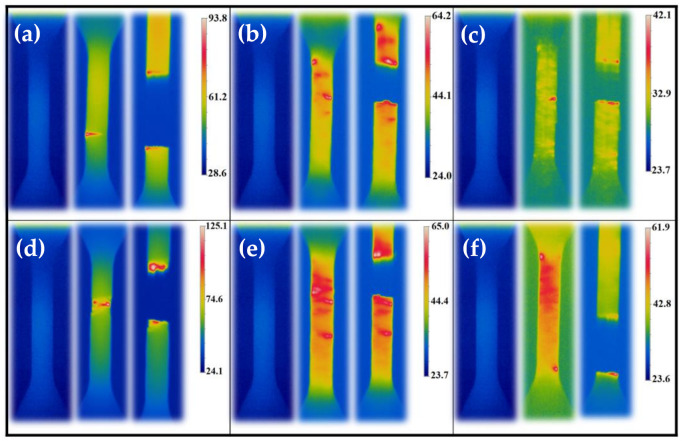
Induced temperature under tension–tension fatigue test for condition III-1 at (**a**) σ = 24 MPa, (**b**) σ = 40 MPa, and (**c**) σ = 52 MPa; and condition III-2 at (**d**) σ = 24 MPa, (**e**) σ = 40 MPa, and (**f**) σ = 52 MPa for frequency of 80 Hz.

Figure 10 illustrates the temperature variation at different amplitudes for samples printed according to condition III-3. As can be seen, the temperature enhancement was shown from the start of the test until the failure of the sample. These illustrations clearly show how induced temperature during the fatigue test increased until the failure of the material. In the case of other conditions, Figure 11 shows the induced temperature at some instances during the tension–tension fatigue test. From the thermogram indicated in Figure 11, one can note that the influence of process parameters is evident. As mentioned in the previous sections, keeping the temperature profile of filaments around or above crystallization temperature allows the material to have more time to be crystallized, and this issue causes better strength between the interface of filaments [16].

**Figure 12:**
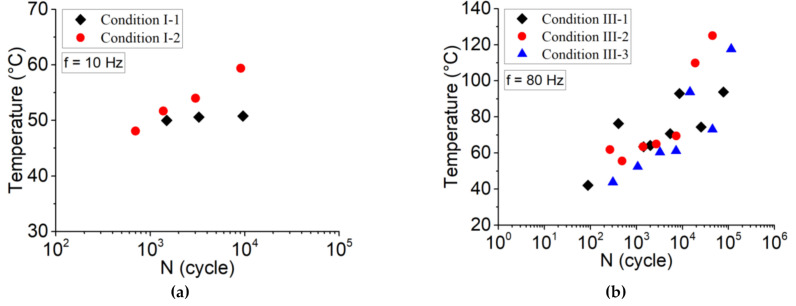
Maximum induced temperature evolution versus number of cycles according to (**a**) condition I at frequency of 10 Hz and (**b**) condition III at frequency of 80 Hz.

Considering temperature evolution as a critical parameter in fatigue lifetime, maximum induced temperature can be a factor in predicting the fatigue behavior of the material. Figure 12 illustrates the maximum induced temperature (just before failure). The effect of frequency shows that, at 10 Hz, temperature increased around the glass transition temperature, while for 80 Hz, temperature increased around crystallization temperature of the material. One can conclude that both fatigue behavior and fatigue life of PLA-Graphene 3D-printed composites are influenced by a coupled effect of loading amplitude and frequency, which induced thermal effect and progressive damage. In fact, the analysis of the self-heating phenomenon together with the evolution of the relative Young’s modulus can be a good way to separate thermal fatigue and MF.

**Figure 13:**
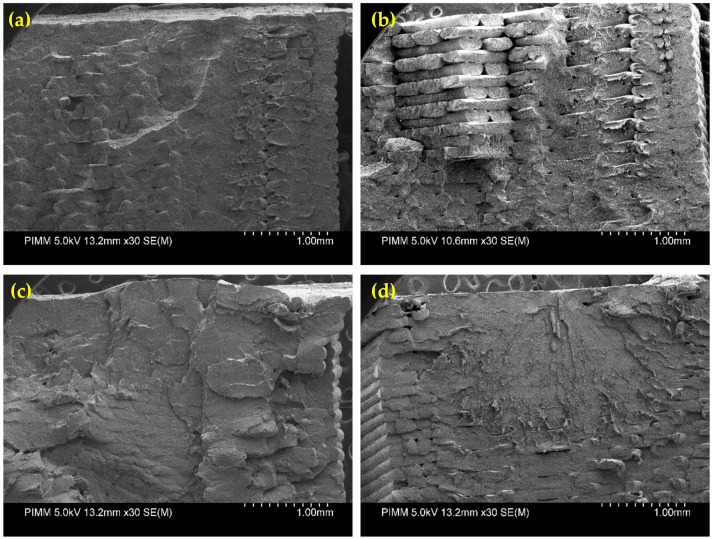
SEM micrograph for fracture surface during fatigue test of the specimen in condition I-1: (**a**) σ = 24 MPa–f = 10 Hz and (**b**) σ = 52 MPa–f = 10 Hz; and in condition III-1: (**c**) σ = 24 MPa–f = 80 Hz and (**d**) σ = 52 MPa–f = 80 Hz.

For emphasizing the coupled effect of loading amplitude and frequency at microscopic scale fracture, surface observations were performed. Figure 13 compares the fracture surfaces of printed samples obtained for two applied stresses at the frequencies of 10 and 80 Hz. The analysis on the SEM images highlights the following remarks:

- The fracture surface observed in the case of low frequency (Figure 13a,b) shows that the enhancement of the induced temperature until glass transition temperature at low amplitude tends to increase the ductility of the sample, whereas the brittle fracture is more obvious at a high amplitude.
- SEM observations on the samples tested at 80 Hz highlight ductile fracture (Figure 13c,d). In fact, self-heating provides more deformation at low amplitude; however, the influence of high frequency and the ductile behavior of the sample is inevitable.
- In general, the higher the self-heating, the more the ductility of the material.

## 4. Conclusions

The mechanical behavior of 3D-printed PLA-Graphene specimens under both quasi-static and fatigue loadings is influenced mainly by the different sources of temperature and printing speeds.

In this paper, the mechanical properties of PLA-Graphene specimens were investigated by considering the effect of platform temperature and print speed (liquefier temperature was fixed at T = 200 °C). A strong variation of fatigue lifetime, as a function of the loading amplitude, frequency, and the mentioned parameters, is presented. As observed from the obtained results, fatigue lifetime clearly depends on the mentioned process parameters. Moreover, when the frequency is 80 Hz, self-heating decreases the fatigue lifetime, which also depends on the coupling effect of thermal and mechanical fatigue. SEM micrographs demonstrated that ductile behavior was observed for the samples tested at frequency of 80 Hz, whereas the polymer remained almost brittle during the fatigue tests at 10 Hz.

## Figures and Tables

**Table 1:** Characteristics of the applied PLA-Graphene filament (adapted from manufacturer datasheet, FILOALFA, Italy).

Properties	Typical Value
Material Density	1.24 g/cm^3^
Diameter (Tolerance)	1.75 mm (± 0.01 mm)
Glass Transition Temperature	72 °C
Melting Temperature	158 °C

**Table 2:** Representation of chosen parameters for 3D printing of PLA-Graphene samples.

Printing Parameters	Value
Nozzle temperature (°C)	200
Platform temperature (°C)	40–50–60
Chamber temperature (°C)	30
Print speed (mm/s)	20–30–40
Layer height (mm)	0.15
Infill density (%)	100
Infill pattern	line
Number of bottom/top layers	2/2
Number of contours (wall)	2
Infill line directions (relative to the long axis of the test bar) (°)	(45/−45)

**Table 3:** Various conditions of printing.

Condition		Liquefier Temperature (°C)	Platform Temperature (°C)	Print Speed (mm/s)
I	1	200	40	20
	2		50	
	3		60	
II	1	200	40	30
	2		50	
	3		60	
III	1	200	40	40
	2		50	
	3		60	

**Table 4:** Results of the tensile behavior of printed samples according to conditions I, II, and III.

Condition		E (GPa)	$\sigma_{max}\;(\mathrm{MPa})$	$\varepsilon \;at\;\sigma_{max}\;(\%)$
I	1	1.8 ± 0.1	59 ± 1	3.8 ± 0.2
	2	2.1 ± 0.1	60 ± 1	3.9 ± 0.2
	3	2.1 ± 0.1	62 ± 1	4 ± 0.2
II	1	2.1 ± 0.1	61 ± 1	3.9 ± 0.2
	2	2.2 ± 0.1	61 ± 1	3.9 ± 0.2
	3	2.2 ± 0.1	63 ± 1	4.2 ± 0.2
III	1	2.2 ± 0.1	62 ± 1	4.1 ± 0.2
	2	2.3 ± 0.1	63 ± 1	4.2 ± 0.2
	3	2.4 ± 0.1	65 ± 1	4.4 ± 0.2

## Data Availability

The data presented in this study are available on request from the corresponding author.
